# Medicoeconomic Index for Photo-Induced Skin Cancers

**DOI:** 10.1155/2012/414683

**Published:** 2011-11-01

**Authors:** Ioana Chiorean, Liana Lupşa, Luciana Neamţiu, Maria Crişan

**Affiliations:** ^1^Faculty of Mathematics and Computer Science, Babeş-Bolyai University, 400084 Cluj-Napoca, Romania; ^2^Department of Epidemiology and Biostatistics, Cancer Institute Ion Chiricuţă, 40445 Cluj-Napoca, Romania; ^3^Department of Histology, University of Medicine and Pharmacy “Iuliu Haţieganu”, Cluj- Napoca, Romania

## Abstract

Like in every type of cancer, in skin cancer the efficiency of the medical treatment is very important. In the present paper, a Bayesian model for the management of this disease is given, and a medical index to measure the effectiveness of treatment from medical, economical, and quality of life point of view is presented, taking into account some of the patients characteristics.

## 1. Introduction

During the last 10–15 years an alarming increase of the photo-induced cutaneous cancers was registered nationally and internationally (1.000.000 new cases in 2007 in the USA), reaching epidemic proportions in some areas. At the moment, the number of patients with cutaneous cancers has significantly increased in Romania. The real incidence of this pathology is unknown, partly due to the lack of an integrated national or regional database. The phenomenon is more frequent in the case of persons who uncontrollably expose themselves to ultraviolet rays for cosmetic or therapeutic reasons or due to improper work conditions, without protection outfits, but it depends also on other reasons, as photo type, age, gender, and so forth. Since we witness a continuous process of ozone layer alteration, global warming, and increase in the pollution degree, the prevention of photo-induced cutaneous cancers and elaboration of protocols for precocious diagnosis and innovative treatment appear as a priority issue of public health (see [1]).

One strategy used in medicine to identify and treat a disease is by screening. Its purpose is to identify in an early stage some malady in a community, enabling timely intervention and management in order to reduce suffering and mortality. In the specific case of skin cancer, this strategy also functions. In order to obtain good results, the measurements have to be taken from different points of view. For instance, most published researches are based on the study of cost and effectiveness of a treatment (see [2–6]), taking into account different characteristics of the patients. The present paper proposes to determine the efficiency of the treatment also from the quality of life point of view. The results are obtained by means of a Bayesian approach for determining the involved parameters, and, as a measure, the economical index is obtained.

## 2. Bayesian Regression Model

We consider a sample of *n* individuals with melanoma in different stages, participating in a clinical trial and denote by (*Ef*_*i*_) the effectiveness data, by (*C*_*i*_) cost data, and by (*Q*_*i*_) the quality of life for each patient *i*, *i* ∈ {1,…, *n*}. These *n* patients are considered to be split in two different groups: those who are under treatment and those who are not. The results of the clinical trial, in terms of effectiveness, costs, and quality of life, are not determined only by the type of treatment received, and so it is necessary to consider a series of possible covariates that may influence the above results. Such covariates include the patient's photo type, age, state of health at the time of the clinical trial, gender, and other characteristics that depend on the type of clinical trial under analysis. We define *X* as an *n* × (*k* + 1) matrix of covariates, where each column (*X*_*i*_) refers to one covariate. The first column is a column of ones referring to the constant (see [7, 8]). We seek, therefore, to explain the results obtained (*Ef*_*i*_, *C*_*i*_, and *Q*_*i*_), as a linear combination of the *k* covariates considered (the patient's individual characteristics and the type of treatment received). For this purpose, we propose a Bayesian multiple linear regression model in which the perturbation terms (*u*_*i*_, *v*_*i*_, and *w*_*i*_) are assumed to be Gaussian, independent, and identically distributed with a mean of 0 and variances of *σ*_1_^2^, *σ*_2_^2^, and *σ*_3_^2^, respectively,

(1) $$\begin{array}{c} Ef_{i}=\beta_{0}+\beta_{1}\cdot X_{1,i}+\beta_{2}X_{2,i}+\cdots +\beta_{k-1,i}+\beta_{T,i}+u_{i},\\ C_{i}=\delta_{0}+\delta_{1}\cdot X_{1,i}+\delta_{2}X_{2,i}+\cdots +\delta_{k-1,i}+\delta_{T,i}+v_{i},\\ Q_{i}=q_{0}+q_{1}\cdot X_{1,i}+q_{2}X_{2,i}+\cdots +q_{k-1,i}+q_{T,i}+w_{i}, \end{array}$$

where the vectors *β* = (*β*_0_, *β*_1_,…, *β*_*k*−1_, *β*_*T*_), *δ* = (*δ*_0_, *δ*_1_,…, *δ*_*k*−1_, *δ*_*T*_), *q* = (*q*_0_, *q*_1_,…, *q*_*k*−1_, *q*_*T*_), and the accuracy values *τ*_1_ = *σ*_1_^−2^, *τ*_2_ = *σ*_2_^−2^, *τ*_3_ = *σ*_3_^−2^ are the parameters of the model.

The *k* covariates considered for which data are available need not to be explicative of the effectiveness, the costs, and the quality of life, and so the above general model could be corrected by eliminating those covariates that do not explain effectiveness and cost.

The first step to be taken in estimating the parameters is to determine the likelihood function of effectiveness *l*_*e*_(*Ef* | *β*, *τ*_1_), *l*_*c*_(*C* | *δ*, *τ*_2_), *l*_*q*_(*Q* | *q*, *τ*_3_), where *Ef* = (*Ef*_1_,…, *Ef*_*n*_), *C* = (*C*_1_, …, *C*_*n*_), and *Q* = (*Q*_1_,…  , *Q*_*n*_). In this stage both costs, effectiveness and qualities of life are assumed to present a normal distribution, and so the likelihood functions are represented by the following expressions:

(2) $$\begin{array}{cc} & l\left(Ef,C,Q\;|\;\beta ,\delta ,q,\tau_{1},\tau_{2},\tau_{3}\right)\\ & \;=l_{e}\left(Ef\;|\;\beta ,\tau_{1}\right)\cdot l_{c}\left(C\;|\;\delta ,\tau_{2}\right)\cdot l_{q}\left(Q\;|\;q,\tau_{3}\right), \end{array}$$

where

(3) $$\begin{array}{c} l_{e}\left(Ef\;|\;\beta ,\tau_{1}\right)\propto \tau_{1}^{N/2}\cdot \exp\left\{\frac{\tau_{1}}{2}{\left(Ef-X\beta \right)}^{\prime }\left(Ef-X\beta \right)\right\},\\ l_{c}\left(C\;|\;\delta ,\tau_{2}\right)\propto \tau_{2}^{N/2}\cdot \exp\left\{\frac{\tau_{2}}{2}{\left(C-X\delta \right)}^{\prime }\left(C-X\delta \right)\right\},\\ l_{q}\left(Q\;|\;q,\tau_{3}\right)\propto \tau_{3}^{N/2}\cdot \exp\left\{\frac{\tau_{3}}{2}{\left(Q-Xq\right)}^{\prime }\left(Q-Xq\right)\right\}. \end{array}$$

Assuming model (1) from a Bayesian point of view, we must specify the prior distribution for the 3 · *k* + 6 parameters of the model. The prior distribution represents expert information about the set of model parameters before the sample observations are analysed. We propose a normal (*N*), respectively, gamma (*G*) form for the base prior and assume independence between the coefficients *β*, *δ*, *q* and precision terms *τ*_1_, *τ*_2_, *τ*_3_. Therefore,

(4) $$\begin{array}{c} \pi \left(\beta ,\tau_{1}\right)=\pi_{e,1}\left(\beta \right)\cdot \pi_{e,2}\left(\tau_{1}\right),\\ \pi \left(\delta ,\tau_{2}\right)=\pi_{c,1}\left(\delta \right)\cdot \pi_{c,2}\left(\tau_{2}\right),\\ \pi \left(q,\tau_{1}\right)=\pi_{q,1}\left(q\right)\cdot \pi_{q,2}\left(\tau_{3}\right), \end{array}$$

where

(5) $$\begin{array}{c} \pi_{e,1}\left(\beta \right)\approx N\left(\beta_{0},V_{1}^{-1}\right),\;\;\pi_{c,1}\left(\delta \right)\approx N\left(\delta_{0},V_{2}^{-1}\right),\\ \pi_{q,1}\left(q\right)\approx N\left(q_{0},V_{3}^{-1}\right),\\ \pi_{e,2}\left(\tau_{1}\right)\approx G\left(a_{1},b_{1}\right),\;\;\pi_{c,2}\left(\tau_{2}\right)\approx G\left(a_{2},b_{2}\right),\\ \;\;\pi_{q,2}\left(\tau_{3}\right)\approx G\left(a_{3},b_{3}\right). \end{array}$$

The parameters *β*_0_, *V*_1_, *δ*_0_, *V*_2_, *q*_0_, *V*_3_, *a*_1_, *b*_1_, *a*_2_, *b*_2_, *a*_3_, *b*_3_, which determine the prior distribution, are defined on the basis of the initial information (available when the analysis begins). The joint posterior distribution of the parameters *β*, *δ*, *q*, *τ*_1_, *τ*_2_, *τ*_3_, given the data (*Ef*, *C*, *Q*), can be calculated from (4), using Bayes' theorem

(6) $$\begin{array}{cc} & \pi \left(\beta ,\tau_{1}\;|\;Ef\right)\propto \tau_{1}^{(n+2a_{1})/2-1}\\ & \;\cdot \exp\{-\frac{1}{2}[{\left(Ef-X\beta \right)}^{\prime }\left(Ef-X\beta \right)\;\;\\ & \;\;\;\;\;\;+\left(\beta -\beta_{0}\right)V_{1}^{-1}\left(\beta -\beta_{0}\right)+2b_{1}\tau_{1}]\},\\ & \pi \left(\delta ,\tau_{2}\;|\;C\right)\propto \tau_{2}^{(n+2a_{2})/2-1}\\ & \;\cdot \exp\{-\frac{1}{2}[{\left(C-X\delta \right)}^{\prime }\left(C-X\delta \right)\;\;\\ & \;\;\;\;\;\;+\left(\delta -\delta_{0}\right)V_{2}^{-1}\left(\delta -\delta_{0}\right)+2b_{2}\tau_{2}]\},\\ & \pi \left(q,\tau_{3}\;|\;Q\right)\propto \tau_{3}^{(n+2a_{3})/2-1}\\ & \;\cdot \exp\{-\frac{1}{2}[{\left(Q-Xq\right)}^{\prime }\left(Q-Xq\right)\;\;\\ & \;\;\;\;\;\;+\left(q-q_{0}\right)V_{3}^{-1}\left(q-q_{0}\right)+2b_{3}\tau_{3}]\}. \end{array}$$

Inferences about quantities of interest must be based on these posterior distributions. The prior distributions used here are not the only possible choices.

## 3. The Medicoeconomical Evaluation

The standard measure used to compare only the cost and effectiveness of treatments is the incremental cost-effectiveness ratio (ICER). Nevertheless, this measure presents severe interpretational problems, as well as difficulties in estimating the confidence or credibility intervals. The incremental net benefit (INB) has been proposed as an alternative to ICER (see [8]). The INB of treatment 1 (new) versus treatment 0 (actual or control) is defined as

(7) $$\text{INB}\left(R_{c}\right)=R_{c}\left(\mu_{1}-\mu_{2}\right)-\left(\nu_{1}-\nu_{2}\right),$$

where *μ*'s and *ν*'s are the mean effectiveness and cost of the respective treatments. The value *R*_*c*_ is interpreted as the cost that decision-makers are willing to accept in order to increase the effectiveness of the treatment applied by one unit. Thus, analyzing whether the alternative treatment is more cost effective than the control treatment is equivalent to determining whether INB(*R*_*c*_) is positive.

For our problem, we use the medicoeconomical index denoted by MEI(*λ*_*e*_, *λ*_*c*_, *λ*_*q*_) introduced in [3], according to

(8) $$\text{MEI}\left(\lambda_{e},\lambda_{c},\lambda_{q}\right)=\lambda_{e}\left(\mu_{1}-\mu_{0}\right)+\lambda_{c}\left(\nu_{1}-\nu_{0}\right)+\lambda_{q}\left(\gamma_{1}-\gamma_{0}\right),$$

where *μ*'s, *ν*'s, and *γ*'s are the mean effectiveness, cost, and quality of life of the respective treatments. The values *λ*_*e*_, *λ*_*c*_, and *λ*_*q*_ are weights (0 ≤ *λ*_*e*_ ≤ 1, 0 ≤ *λ*_*c*_ ≤ 1, 0 ≤ *λ*_*q*_ ≤ 1, *λ*_*q*_ + *λ*_*q*_ + *λ*_*q*_ = 1) which reflect the importance we give to each criteria (the medication effect, the medication costs, the quality of life).
